# Genome-Wide Identification and Expression Profiling Analysis of the *Aux/IAA* Gene Family in *Medicago truncatula* during the Early Phase of *Sinorhizobium meliloti* Infection

**DOI:** 10.1371/journal.pone.0107495

**Published:** 2014-09-16

**Authors:** Chenjia Shen, Runqing Yue, Yanjun Yang, Lei Zhang, Tao Sun, Luqin Xu, Shuanggui Tie, Huizhong Wang

**Affiliations:** 1 College of Life and Environmental Sciences, Hangzhou Normal University, Hangzhou, China; 2 Henan Academy of Agricultural Sciences, Zhengzhou, China; 3 Department of Plant Pathology, Washington State University, Pullman, Washington, United States of America; University of Nottingham, United Kingdom

## Abstract

**Background:**

Auxin/indoleacetic acid (*Aux/IAA*) genes, coding a family of short-lived nuclear proteins, play key roles in wide variety of plant developmental processes, including root system regulation and responses to environmental stimulus. However, how they function in auxin signaling pathway and symbiosis with *rhizobial* in *Medicago truncatula* are largely unknown. The present study aims at gaining deeper insight on distinctive expression and function features of *Aux/IAA* family genes in *Medicago truncatula* during nodule formation.

**Principal Findings:**

Using the latest updated draft of the full *Medicago truncatula* genome, a comprehensive identification and analysis of *IAA* genes were performed. The data indicated that *MtIAA* family genes are distributed in all the *M. truncatula* chromosomes except chromosome 6. Most of *MtIAA* genes are responsive to exogenous auxin and express in tissues-specific manner. To understand the biological functions of *MtIAA* genes involved in nodule formation, quantitative real-time polymerase chain reaction (qRT-PCR) was used to test the expression profiling of *MtIAA* genes during the early phase of *Sinorhizobium meliloti* (*S. meliloti*) infection. The expression patterns of most *MtIAA* genes were down-regulated in roots and up-regulated in shoots by *S. meliloti* infection. The differences in expression responses between roots and shoots caused by *S. meliloti* infection were alleviated by 1-NOA application.

**Conclusion:**

The genome-wide identification, evolution and expression pattern analysis of *MtIAA* genes were performed in this study. The data helps us to understand the roles of MtIAA-mediated auxin signaling in nodule formation during the early phase of *S. meliloti* infection.

## Introduction

The phytohormone auxin plays essential roles during the entire life-cycle of plants [1], [2]. Indole-3-acetic acid (IAA), the primary auxin in higher plants, has been found to effect on regulating diverse aspects of plant growth and development under environmental stimuli responses [3]. Some gene families, including *Aux/IAA*, *GH3* (*Gretchen Hagen3*) and *SAUR* (*small auxin up RNA*), are responsive to auxin stimulation during early stage of auxin signaling transduction [4]. To be an important component of auxin signaling pathway, auxin/indole-3-acetic acid (Aux/IAA) proteins were well known as a direct target of the auxin transport inhibitor response 1 (TIR1) and its paralogs AUXIN RECEPTOR F-BOX/AFBs [5], [6]. Dynamic auxin concentration leads to the degradation of Aux/IAA proteins, which were involved in expression regulation of many auxin response genes by releasing ARFs (Auxin Response Factor) [7]–[9].

Most of Aux/IAA proteins contain four highly conserved domains: I, II, III and IV. Domain I, which contains a conserved leucine repeat (LXLXLX) motif, interacts with TOPLESS (TPL) protein to mediate auxin-dependent transcriptional repression during embryogenesis [10]. Domain II is responsible for the instability of Aux/IAA proteins [11]. Domain III and IV are the binding sites for homo- and hetero-dimerization among the Aux/IAA proteins and ARF proteins [12], [13]. It was reported that domain III and IV function as a complex. The crystal structure analysis revealed that Bem1p (PB1) domain in C-terminal is the special domain for the protein-protein interaction between Aux/IAA and ARF [14]. Moreover, Aux/IAA proteins contain two nuclear localization signals (NLS), which locate Aux/IAA proteins to the nucleus [15], [16].

In the past years, the functions of *Aux/IAA* family genes have been well studied. In *Arabidopsis*, loss function of IAA1/AXR5, which is a substrate of SCF (TIR1), causes a variety of auxin-related growth defects and auxin insensitivity phenotype [17]; *iaa3/shy2* loss-of-function mutation affects auxin homeostasis and formation of lateral roots [18]; *iaa7/axr2*, *iaa17/axr3*, *iaa19/msg2* and *iaa28* are involved in the reduction of lateral root number [19]–[22]; *iaa14/slr* mutant even completely blocks lateral root formation [23]; a gain-of-function mutant *iaa16* impedes plant growth and confers decreased response to phytohormone [20]. In monocot rice, some *IAA* genes also have been reported. Over-expression of *OsIAA1* leads to inhibition of root elongation and shoot growth [24]; a gain-of-function in OsIAA11 results in the absent of lateral roots [25]; OsIAA23 defines postembryonic maintenance of quiescent center (QC) in rice [26]; OsIAA31 functions in lateral root development [27].

Many legume species including *M. truncatula* interact with nitrogen fixing bacteria (rhizobia) to form nodules, which are the symbiotic organ of legumes to host nitrogen-fixing bacteria [28], [29]. Phytohormone auxin has been reported to be a crucial element involved in nodules formation in the *M. truncatula* roots [30], [31]. As we already know, there is a close relationship between auxin signaling and rhizobia stimulation during the symbiosis formation [32]. As a model indeterminate legume, *M. truncatula* is used to reveal how auxin early responsive genes participate in nodule initiation and formation. The main role of auxin in regulation of mitosis during nodulation is controlling the inner and out cortical cell dividing [30]. After rhizobia stimulate, auxin signaling transduction and endogenesis auxin transport from shoots to roots are essential processes for root system architecture (RSA) regulation, which enhances pseudonodules formation on rhizobia inoculation roots [30], [33]. However, the underlying mechanism how auxin early responsive *Aux/IAA* family genes are involved in nodule formation at an early phase of *S. meliloti* infection of *M. truncatula* remains largely unknown

As an important part of auxin signaling pathway, Aux/IAAs are encoded by a multigene family and exist in most plant species. There are 29 members in *Arabidopsis*, 26 members in tomato (*Solanum lycopersicon*), 34 members in maize (*Zea mays L*.), and 31 members in rice (*Oryza sativa L*.) and 27 members in cucumber (*Cucumis sativus L.*) [16], [27], [34]–[38]. In this work, we provide comprehensive information on the genomic structures, chromosomal locations, sequences homology and expression patterns of seventeen *IAA* genes in *M. truncatula*. Our studies give a new insight into the complexity of *M. truncatula IAA* expressions at an early phase of *S. meliloti* infection. The distinct spatio-temporal expression patterns of *M. truncatula IAA* family genes and their differential responses to rhizobial symbiosis provided clues for functional characterization of these auxin responsive genes involved in formation of root nodules.

## Materials and Methods

### Plant material, growth conditions and hormone treatment

*Medicago truncatula* (Jemalong) A17 was used in this study. Seeds were dipped in sulfuric acid for 10 min to degrade seed coat, and washed with sterilized water three times. Then the seeds were germinated on plates contained 0.8% agar at 25°C until the radicals were about 1 cm. The seedling were grown in large plastic buckets containing full-strength nutrient solution and were incubated in a growth chamber at 22°C constantly during a 16 hour day and 8 hour night with a photon flux density of 100 µmolm^−2^s^−1^ for 14 days. The composition of full-strength nutrient solution is: 0.25 mM KH_2_PO_4_, 0.5 mM MgSO_4_·7H_2_O, 0.125 mM CaCl_2_, 1.25 mM KNO_3_, 0.5 mM NH_4_NO_3_, 15 µM H_3_BO_3_, 2.5 µM MnSO_4_·H_2_O, 0.5 µM ZnSO_4_·7H_2_O, 0.5 µM CuSO_4_·5H_2_O, 0.35 µM NaMoO_4_·2H_2_O, and 50 µM Fe (III) EDTA with a pH of 6.0. In auxin response experiment, the IAA concentration was 0.1 µM. Samples of shoots and roots were used to test the changes of *MtIAA* genes expression level at different time points (3 hr, 6 hr, 12 hr and 24 hr). The data were analyzed by five independent repeats, and standard deviations were shown with error bars. The 14-day-old seedlings were transferred to a nitrogen-free BNM medium [39] for *S. meliloti* infections and auxin treatment experiments. The seedlings were soaked in liquid BNM medium under different treatments. These treatments are -Sin/-NOA, +Sin/−NOA, −Sin/+NOA and +Sin/+NOA. The treatment –Sin/-NOA was used as mock treatment. (Sin  =  S. meliloti infection; NOA = 10 µM 1-NOA treatment; -Sin/-NOA: mock treatment). Then shoots and roots of *M. truncatula* seedlings were collected for RNA isolation respectively. Experiments were repeated for five biological times.

### Identification of *IAA* genes in *Medicago truncatula*

The hidden Markov model (HMM) profile of the Aux/IAA protein family (Pfam: 02309 AUX/IAA family) was employed to identify the *Aux/IAA* genes from the *M. truncatula* genome. The profile was used to search the complete proteome of *M. truncatula* available in phytozome (http://www.phytozome.net/). All the obtained sequences were sorted as unique sequences for further Aux/IAA domains search using InterProScan Sequence Search (http://www.ebi.ac.uk/Tools/pfa/iprscan/). Linear display of synteny blocks was analyzed by the SyMAP database (http://www.symapdb.org/projects/fabaceae/)

### Phylogenetic tree building, intron/exon structure, genome distribution and motif prediction

Multiple sequence alignments were performed on the MtIAA protein sequences using ClustalW with the default parameters, and the alignments were then adjusted manually. Phylogenetic tree was constructed with aligned MtIAA protein sequences using MEGA5.1 (http://www.megasoftware.net/mega5/mega.html) employing the neighbor-joining (NJ) method. Bootstrap values were calculated from 1000 iterations. The gene pairs displayed high bootstrap value (>99%) were identified as sister pair genes. The constructed tree file was visualized by TreeView1.6 (http://taxonomy.zoology.gla.ac.uk/rod/treeview.html). The software MEGA 5.1 was used for prediction four classical domains in MtIAA proteins (domain I, II, III, IV). The DNA and cDNA sequences corresponding to each predicted gene from the M. truncatula genome and the information of MtIAAs intron distribution pattern were obtained from the). To obtain the gene locations, we drew a map of the distribution of MtIAA genes throughout the M. truncatula genome using genome visualization tool CIRCOS (). *M. truncatula* chromosomes are arranged in a circle and the centromere of each chromosome is marked in black. Ribbon links represent the segmental duplication region retrieved from the SyMAP database. Motifs constitution of *M. truncatula* Aux/IAA proteins were investigated by MEME web server.

### RNA isolation and qRT-PCR

The methods, including RNA extraction from various organs, reverse transcription to cDNA and qRT-PCR analysis, were performed according to Shen's publication[40]. Total RNA from cotyledons, leaves, shoots, roots and flowers was extracted using a Plant RNeasy Mini kit (Qiagen) according to the manufacturer's instructions. Then DNase I treatment was used to remove genomic DNA contamination from total RNA. The primer sequences are listed in Table S1. *Mt-Actin* (*MTR_2g008050*) was used as an internal standard to calculate the relative fold differences based on the comparative *Ct* method. 2^−ΔΔ*Ct*^ refers to the fold difference in *IAA* expression compared with the untreated seedlings. Heat map representation was performed using the normalized *Ct* value with ClustalW software and Treeview to visualize the analysis data.

### Bacterial strains and rhizobia infection

The rhizobia strain used for inoculating the roots of seedlings was *S. meliloti* strain 1021, a streptomycin-resistant derivative of wild-type. *S. meliloti* was grown overnight at 28°C in liquid LBMC medium (10 g/L tryptone, 5 g/L yeast extract, 10 g/L NaCl, 2.6 mM MgSO_4_, 2.6 mM CaCl_2_) supplemented with 200 µg/mL streptomycin, then collected by centrifugation, and suspended in 10 mM MgSO_4_. The bacterial suspension was diluted with liquid BNM medium to OD_600_ = 0.1. For plant inoculation experiment, each seedling was put in petri dish containing nitrogen-free nutrient solution and treated with BNM medium diluted bacteria. For control (mock-inoculated), the seedlings were treated with sterilized BNM medium containing 10 mM MgSO_4_ without bacteria. Five different sets of plants were grown and used for the various analyses.

### Analysis of AuxRE *cis*-elements

The promoters (−1000 to −1 bp before ATG) of *MtIAA* genes were scanned using Watson and Crick words for AuxRE related *cis*-element 1 (AUX1: TGTCTC core sequence), a less stringent variant called AUX2 (TGTVYS), three different bZIP Response Elements (ZREs) (bZIP-associated G-box Related Element (GRE): BACGTV, TGA: TGACG and AC-motif: ACTCAT) and two Myb Response Elements (MREs) (MRE1: AMCWAMC and MRE2: GGWTW). Different colors represent different *cis*-elements, which were added to the image manually. The promoter sequences were obtained from http://www.phytozome.net/.

## Results

### Genome-wide identification of *IAA* genes in *Medicago truncatula*

The new version (V3.5) of the *M. truncatula* genome and protein sequences was obtained from the *M. truncatula* genome database. Comprehensive searches of public genomic database using the hidden Markov model (HMM) profile of the *IAA* gene family (Pfam 02309: AUX/IAA family) was employed to identify the *IAA* genes selected from BLAST results. All the candidate Aux/IAA protein sequences were aligned by ClustalW and were checked manually. After protein domain and gene structure analysis, seventeen *IAA* genes were identified in *M. truncatula*. The information of these seventeen *MtIAA* genes, including gene names, locus IDs, intron numbers, ORF lengths, chromosome locations and basic parameters of deduced polypeptide, were listed in Table 1. The size of the deduced MtIAA proteins varies markedly ranging from 142 amino acids (MtIAA14) to 537 amino acids (MtIAA9), the corresponding molecular mass varies from 15.99 kDa to 62.45 kDa, and the predicted isoelectric point widely varies from 5.18 (MtIAA14) to 8.93 (MtIAA7). It suggested that different MtIAA proteins might function in different microenvironments.

**Table 1:** MtIAA gene family in Medicago truncatula.

Gene	locus ID	ORF length	No. of introns	Chr no.	Chr location	Deduced polypeptide		
						Length (aa)	Mol wt (kDa)	pI
MtIAA1	Medtr1g070830	615	2	1	17318116–17319328	204	22.91	6.42
MtIAA2	Medtr1g093240	711	4	1	26193287–26196287	236	25.72	8.46
MtIAA3	Medtr1g093350	537	1	1	26247246–26248612	178	19.98	8.00
MtIAA4	Medtr2g100780	810	4	2	32192283–32195676	269	29.96	8.73
MtIAA5	Medtr2g101500	981	4	2	32562344–32565049	326	35.36	7.96
MtIAA6	Medtr2g102490	597	4	2	33070172–33071309	198	22.5	8.62
MtIAA7	Medtr3g106850	642	3	3	37913558–37914702	213	24.73	8.94
MtIAA8	Medtr4g060470	1086	2	4	18656418–18658053	361	39.46	5.93
MtIAA9	Medtr4g115070	1614	4	4	39660114–39669519	537	62.45	6.93
MtIAA10	Medtr4g124300	555	3	4	43252133–43253003	184	20.88	6.83
MtIAA11	Medtr4g128070	522	1	4	44708010–44709260	173	19.22	7.91
MtIAA12	Medtr5g030710	1008	6	5	12694370–12699892	335	36.26	8.25
MtIAA13	Medtr5g067350	1044	5	5	27469789–27473618	335	36.26	8.25
MtIAA14	Medtr7g110790	429	2	7	35419184–35419916	142	15.99	5.18
MtIAA15	Medtr8g014520	816	4	8	3132788–3136332	271	30.12	8.19
MtIAA16	Medtr8g067530	1071	7	8	17235080–17239117	356	38.67	6.62
MtIAA17	Medtr8g103030	882	4	8	30131713–30134764	293	31.89	7.85

### Chromosomal distribution and gene structure analysis of *MtIAA* genes

**Figure 1:**
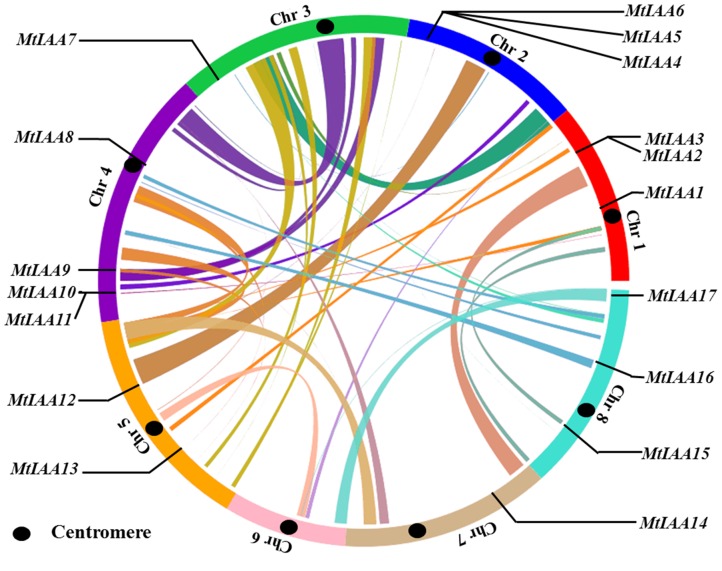
Chromosome mapping of *MtIAA* family genes. The genome visualization tool CIRCOS was employed. *Medicago truncatula* chromosomes are arranged in a circle, and the centromere of each chromosome is marked in black. Ribbon links represent the segmental duplication region retrieved from the SyMAP database. *MtIAA* family genes are mapped by locus.

**Figure 2:**
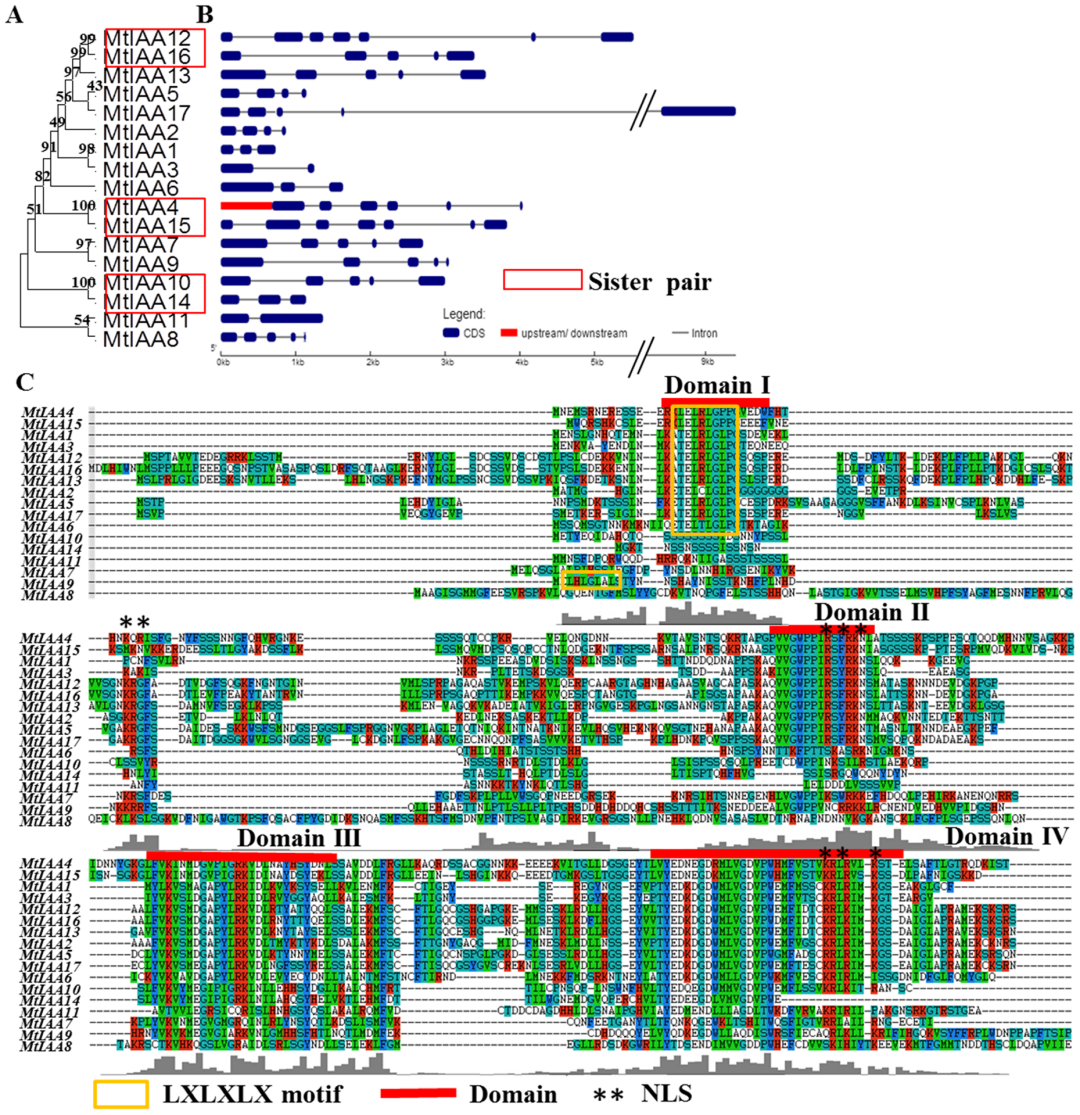
Phylogenetic relationships, exon-intron structure and protein domain analysis of *MtIAA* family genes. (A) An unrooted phylogenetic tree was constructed using ClustalW by N-J method. The sister pair genes are indicated by red boxes. (B) Exon-intron structure analysis of *MtIAA* genes. The untranslated regions (UTRs) are indicated by thick red lines; the exons are indicated by blue boxes; the introns are indicated by gray lines. (C) Alignment of *Medicago truncatula* Aux/IAA proteins obtained with the ClustalW program and manual correction. Multiple alignments of the domains I–IV of the *M. truncatula* Aux/IAA proteins also were showed by red lines. Colorized shading indicates identical and conversed amino acid residues, respectively. The LXLXLX motif was also marked by thin yellow box. Two NLSs were marked by black asterisks.

All seventeen *MtIAA* genes in *M. truncatula* are distributed on seven chromosomes, except for chromosome 6. The distribution of the *MtIAA* genes varies: there are three *MtIAA* genes on chromosome 1, 2 and 8; just a single *MtIAA* gene on chromosome 3 and 7; four *MtIAA* genes on chromosome 4; two *MtIAA* genes on chromosome 5 (Figure 1 and Table 1). The full-length cDNA sequences and genomic DNA sequences of *MtIAA* family genes were downloaded from phytozome 9.1 database (http://www.phytozome.net). The numbers and positions of exons-introns for each *MtIAA* genes were uncovered by a comparison of the full-length cDNA sequences with the corresponding genomic DNA sequences. The number of introns varied from 1 to 6 in the *MtIAA* gene family (Figure 2B). The *MtIAA* genes displayed complex distribution patterns of introns-exons even within the same phylogenetic group.

Gene divergence and duplication events were the important causes for evolutionary momentum [41], [42]. Family genes duplication events including tandem and segmental duplications were used to reveal the expansion of *M. truncatula IAA* family genes during the evolutionary process. In this study, three sister-gene pairs have been showed in Figure 2A and these sister-gene pairs were: *MtIAA12*/*MtIAA16*, *MtIAA4*/*MtIAA15* and *MtIAA10*/*MtIAA14.*

### Protein structure and phylogenetic relationship analysis of MtIAAs

Most of the MtIAA family proteins contain four conserved domains: domain I, II, III and IV (Figure 2C). A typical LXLXLX motif was found in domain I of most MtIAA proteins, including MtIAA1-6, 9, 12, 13, 15, 16 and 17. Two different types of putative nuclear localization signals (NLS) were detected in most MtIAA proteins: a bipartite NLS and a typical NLS. The bipartite NLS contained two stretches of K/R residues. The short amino acid sequence KR is located between domain I and II and the second part of the bipartite NLS is located at the end of domain II. A typical NLS, which consisted of one cluster of largely changed amino acid residues such as lysine or arginine, located at the end of domain IV (Figure 2C) [16], [43].

**Figure 3:**
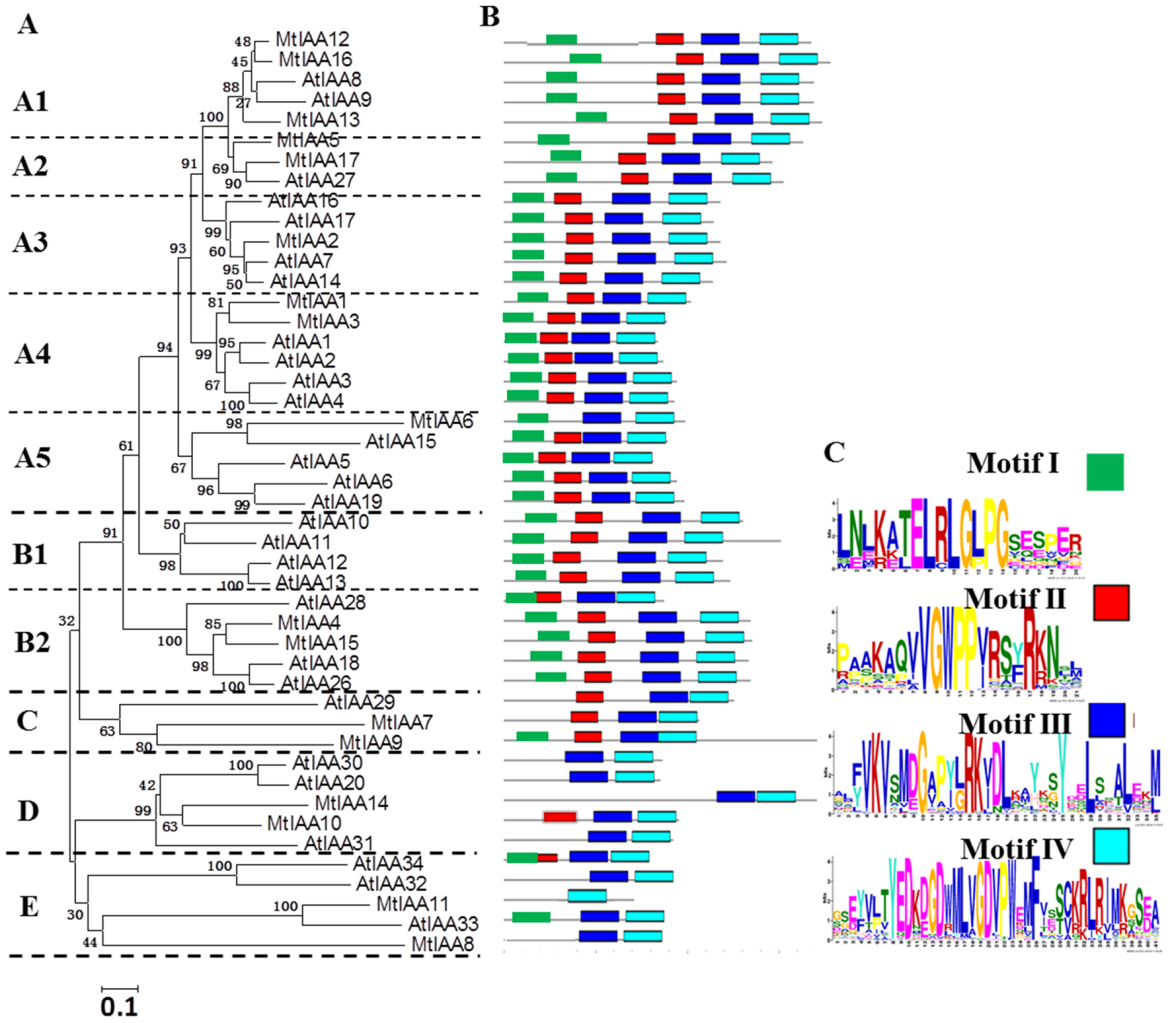
Phylogenetic relationships and motifs distribution analysis. (A) Phylogenetic relationships among *Arabidopsis* and *M. truncatula* IAA proteins. The unrooted tree was generated by MEGA5.1 program by the N-J method. Bootstrap supports from 1000 replicates are showed at each branch. (B) The motif distribution in *Arabidopsis* and *M. truncatula* IAA proteins. Motifs of Aux/IAA proteins were analyzed by MEME web server. Four motifs representing four domains I, II, III and IV were mapped on all Aux/IAA proteins by different colors. (C) The heights of each box represent the specific amino acid conservation in each motif.

To explore phylogenetic relationship among IAA proteins in different plant species, a phylogenetic tree was constructed including IAA family members from *Arabidopsis* and *M. truncatula.* The information of *AtIAA* gene family was listed in Table S3. The unrooted phylogenetic tree was generated by the alignment of full-length protein sequences of seventeen MtIAA proteins and twenty-nine AtIAA proteins [36], [44]. The phylogenetic distribution indicated that IAA proteins classed into five major groups named A, B, C, D and E with well supported bootstrap value. Groups A and B were further divided into several subgroups each: A1, A2, A3, A4 and A5; B1 and B2 (Figure 3A).

The motif distribution in *Arabidopsis* and *M. truncatula* Aux/IAA proteins were analyzed by Multiple Expectation Maximization for Motif Elicitation (MEME) tool (http://meme.nbcr.net/meme/cgi-bin/meme.cgi). Different conserved domains of Aux/IAA proteins were mapped on the Figure 3 by MEME tool. Most IAA proteins, which were belonged to Group A and B, contained four classical IAA domains. The IAA proteins with truncated domains always belonged to subfamilies C, D and E. Motif I cannot be found in MtIAA 7, 8, 10, 11 and 14. Motif II is missing in MtIAA 6, 8, 11 and 14. The domain II of MtIAA10 is modified (DWPPV). MtIAA11 does not display motif III and MtIAA14 is missing a part of domain IV (Figure 3B).

### Expression patterns of *MtIAA* genes in different *M. truncatula* tissues

**Figure 4:**
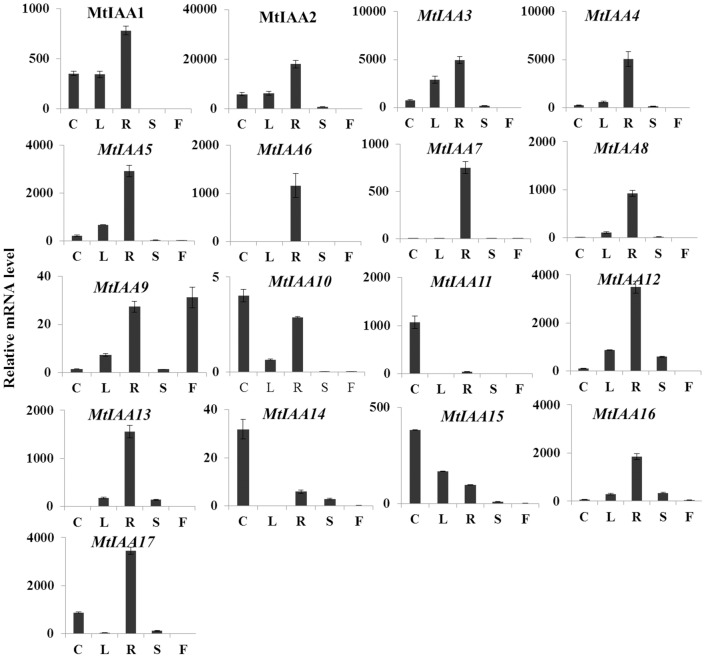
Tissues-specific expression patterns of *MtIAA* genes. Based on the phylogenetic analysis, all *MtIAA* genes were grouped into five subfamilies (A: subfamily A; B: subfamily B; C: subfamily C; D: subfamily D; E: subfamily E). Expression patterns of the *MtIAA* genes in five indicated organs were analyzed by the data of qRT-PCR. The value of ACTIN (Cotyledon)/1000 defines as 1. C: cotyledon; L: leaf; R: root; S: shoot; F: flower. The data were analyzed by five independent repeats, and standard deviations were shown with error bars.

Analysis of transcriptional level of *MtIAA* genes in different *M. truncatula* tissues gives clues on the biological function of these auxin-responsive genes. Here, the spatio specificity expression of each member of the *MtIAA* family gene was examined in different organs including roots, stems, cotyledons and leaves of two weeks seedlings and flowers of two months plants using qRT-PCR. (The primer sequences of seventeen *MtIAA* genes were listed in Table S1). Transcript accumulations of seventeen *MtIAA* genes were detectable in most different organs (Figure 4). The mRNA abundances of *MtIAA* family genes in roots were much higher compared to other organs. Furthermore, the expression levels of *MtIAA* genes in flowers were lower compared to other organs. It suggested that *MtIAA* genes may function in root growth and development. Of particular interest, some *MtIAA* genes showed obviously tissue-specific expression patterns in *M. truncatula*. *MtIAA6* and *MtIAA7* exhibited root-specific expressions; *MtIAA10*, *MtIAA11, MtIAA14* and *MtIAA15* showed a higher expression level in cotyledons than that in other organs.

### Auxin regulation expression of *MtIAA* genes in shoots and roots

**Figure 5:**
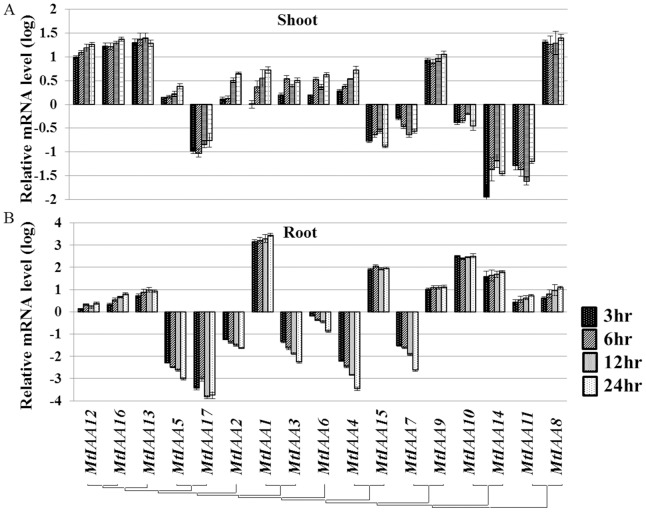
Real-time quantitative RT-PCR (qRT-PCR) analyses of *MtIAA* genes in plants under IAA treatment in both shoots (A) and roots (B). Total RNA was extracted from the shoots and roots of *M. truncatula* seedlings for basal expression. The histogram shows the relative expression level of *MtIAA* genes under IAA treatment compared to the mock expression level. The relative mRNA level of individual genes was normalized with respect to the *MtACTIN* gene. The concentration of synthetic IAA was 0.1 µM. Samples of two different organs (shoots and roots) were used to test the changes of *MtIAA* genes expression level at different time points (3 hr, 6 hr, 12 hr and 24 hr). The data were analyzed by five independent repeats, and standard deviations were shown with error bars.

As an essential compound of auxin signaling pathway, the expression of *IAA* genes showed quick responses to auxin treatment [45]. QRT-PCR was performed with total RNA isolated from shoots and roots of IAA-treated seedlings and mock seedlings. The data showed that most of *MtIAA* genes were responsive to exogenous IAA treatments. The *MtIAA* family genes showed different expression patterns under IAA treatments. The expressions of *MtIAA7*, *MtIAA10*, *MtIAA11*, *MtIAA14*, *MtIAA15* and *MtIAA17* were reduced by IAA treatments and the remaining ones were induced by IAA treatments in shoots (Figure 5A). On the other hand, the expression levels of *MtIAA2-7*, *MtIAA17* were down-regulated by IAA treatments in roots (Figure 5B).

In *M. truncatula*, two opposite auxin-dependent expression patterns were observed under 0.1 µM IAA treatment. While the expression levels of *MtIAA8*, *9*, *12*, *13* and *16* were up-regulated, *MtIAA7* and *17* were down-regulated in both roots and shoots. Many *MtIAA* genes displayed a quick response (3 h treatment) to exogenous auxin application, such as *MtIAA8*, *11*, *12*, *13*, *14* and *16* in shoots and *MtIAA1*, 4, 5, *9*, *10*, *14* and *15* in roots during the time course (Figure 5).

In this work, five sampling time points (0, 3 hr, 6 hr, 12 hr and 24 hr) were used for test whether *IAA* genes in *M. truncatula* were auxin early response genes. The expression levels of most *MtIAA* family genes could be regulated by IAA under 3 hr treatment. The change folds of expression levels were almost the same from 3 hr IAA treatment to 24 hr IAA treatment. Specially, *MtIAA1*, *MtIAA10* and *MtIAA15* were induced by IAA treatment over 100 folds in roots. All the data suggested that most of *MtIAA* family genes were auxin early response genes.

**Figure 6:**
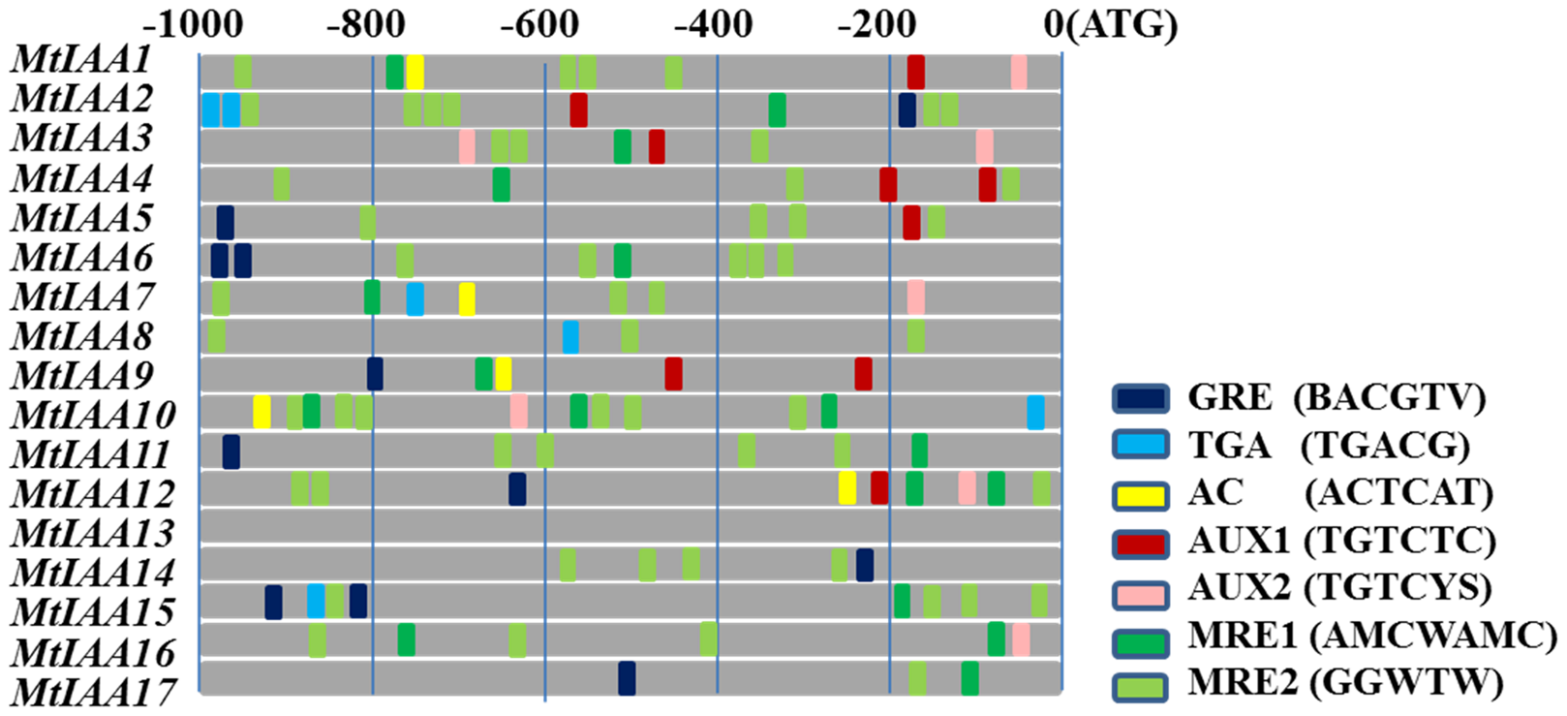
Motif analysis of specific *cis*-elements in promoters of *MtIAA* family genes in *M. truncatula*. The −1000 bp promoter sequences of corresponding *MtIAA* genes were used to analysis of specific ZRE, AuxRE and MRE *cis*-elements, which are given using the presented colour code. Watson and Crick words for AUX1 is TGTCTC; AUX2 is TGTVYS, three different ZREs (GRE: BACGTV; TGA: TGACG; AC-motif: ACTCAT) and two MREs (MRE1: AMCWAMC; MRE2: GGWTW).

The auxin-responsive *cis*-elements in the promoters of *MtIAA* family also were analyzed in this study. The detailed data of this promoter analysis were listed in Table S2. Totally, 11 GREs, 6 TGAs, 5 ACs, 8 AUX1s, 8 AUX2s, 17 MRE1s and 56 MRE2s were contained in all seventeen *MtIAA* promoters (Figure 6).

### Expression analysis of *MtIAA* genes during the early phase of *Sinorhizobium meliloti* infection

Elevation of auxin transport between shoots and roots was the essential process for legume species to response to the rhizobial infection [31]. The expression levels of many auxin-related genes were changed by rhizobial infection during initial infection processes [46]. To reveal how auxin signaling was involved in the nodule formation after rhizobial infection, we examined the expression patterns of *MtIAA* genes in root and shoot of *S. meliloti*-inoculated *M. truncatula* seedlings within 72 hr post inoculation (hpi). First, we germinated the *M. truncatula* wild-type on filter papers, and afterwards transferred the seedlings to liquid medium. Total RNA from roots and shoots were isolated for qRT-PCR analysis. Student's *t*-test analysis between mock-inoculated plants and rhizobial-inoculated plants was performed to reveal the differential expression patterns of *MtIAA* family genes.

**Figure 7:**
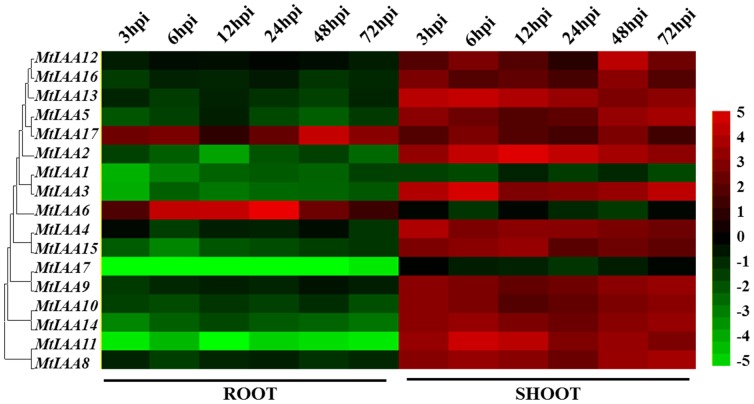
Heat map showing *MtIAA* genes expression pattern at the early phase of *Sinorhizobium meliloti* infection under different conditions. Samples of two different organs (shoots and roots) were used to test the changes of *MtIAA* genes expression level at different time points (3/6/12/24/48/72 hpi) treatment. The different colors correspond to the log-transcription values of the gene change-fold ratio shown in the bar at the right of figure.

Differential expression patterns of *MtIAA* family genes were observed during the early phase of *S. meliloti* infection between shoots and roots in wild-type A17. Most *MtIAA* genes were down-regulated by *S. meliloti* infection in roots and were up-regulated in shoots. However, the expression levels of *MtIAA6* and *MtIAA17* were induced by *S. meliloti* infection in roots; *MtIAA1*, *MtIAA6* and *MtIAA7* were reduced by *S. meliloti* infection in shoots. The main expression pattern of *MtIAA* family genes in roots was opposite to that in shoots (Figure 7).

**Figure 8:**
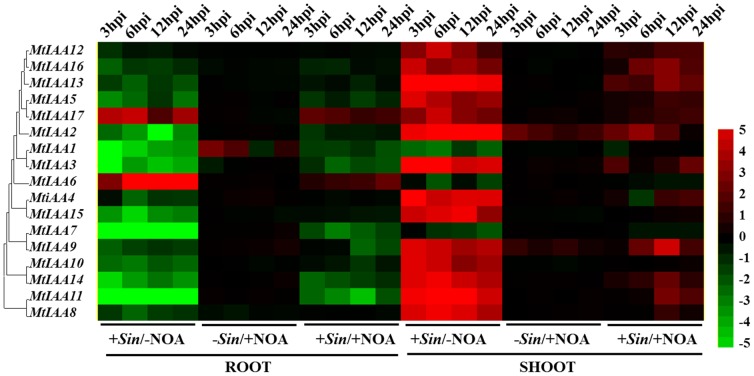
Heat map showing *MtIAA* genes expression pattern at the early phase of *Sinorhizobium meliloti* infection under different conditions. Samples of two different organs (shoots and roots) were used to test the changes of *MtIAA* genes expression level at different time points (3/6/12/24/48/72 hpi) and conditions (−*Sin*/−NOA, +*Sin*/−NOA, −*Sin*/+NOA and +*Sin*/+NOA). The treatment –*Sin*/−NOA was used as mock treatment. The different colors correspond to the log-transcription values of the gene change-fold ratio shown in the bar at the right of figure.

Here, we used 1-NOA (1-naphthoxyacetic acid), that binds to auxin influx transporter to block the auxin polar transport, to suppress the Aux/IAA-mediated auxin signaling in *M. truncatula*. The roots and shoots RNA samples of five time points (0, 3 hpi, 6 hpi, 12 hpi and 24 hpi) were separated into four independent groups: -*Sin*/-NOA, +*Sin*/-NOA, −*Sin*/+NOA and +*Sin*/+NOA. (*Sin*  =  *S. meliloti* infection; NOA = 10 µM 1-NOA treatment; -*Sin*/-NOA: mock treatment). The qRT-PCR data showed that expression levels of most *MtIAA* family genes displayed a drastic decline under +*Sin*/−NOA condition in roots and an obvious increase in shoots. Specially, the −*Sin*/+NOA treatment had no significant effect on expression regulation of *MtIAA* genes. The changes of *MtIAA* genes expression levels caused by *S. meliloti* infection were alleviated by 10 µM 1-NOA application both in roots and shoots (Figure 8).

## Discussion

As a key component of the auxin signaling pathway, Aux/IAA proteins directly bind to ARF proteins and regulate expression of down-stream auxin response genes [47], [48]. Functional characterization and expression analysis of *Aux/IAA* family genes help to reveal the mechanisms behind how auxin signaling is involved in plant growth and responses to environmental changes in a spatio-temporal specific manner. Phytohormone auxin is an important factor in regulation of mitosis, which is involved in nodulation [28], [49], [50]. As a model indeterminate legume, analysis of the protein structure and expression pattern of *Aux/IAA* in *M. truncatula* is a way to elucidate the relationship between auxin signaling and the symbiotic during the early phase of *S. meliloti* infection.

### Characterization and structural analysis of the *MtIAA* family genes in *Medicago truncatula*

Aux/IAA proteins are plant specific transcriptional regulators [51]. In this study, seventeen *MtIAA* family genes were isolated in *M. truncatula* basing on the reference genome sequence (500 Mbp) [52]. The information of seventeen *MtIAA* genes was listed in Table 1. The number of *MtIAA* genes in *M. truncatula* is a little less compared to *Arabidopsis* (29 genes) or rice (31 genes) [27], [36]. Sequence analysis of the *M. truncatula* Aux/IAA family proteins revealed that some MtIAA proteins do not contain all classical domains of this protein family.

Most of MtIAA proteins contain conserved leucine residues in the LXLXLX motif as *A. thaliana* [9]. MtIAA1-6, 9, 12, 13 and 15–17 contain a typical LXLXLX motif “TELRLGLPG”. The LXLXLX motif plays an important role in repression of IAA proteins. Mutation in any of these three Leu in this motif results in total loss of repression or strongly reduced repression in the case of the mutation in the third Leu in the motif [9]. In this study, some MtIAA proteins (5 in 17) do not contain this motif and these MtIAA proteins may function differently to the classical IAA repressors. Four MtIAA proteins do not contain domain II, including MtIAA6, 8, 11 and 14 (Figure 3). There is much evidence that domain II functions as a key motif involved in Aux/IAA protein degradation, it is proposed to be functionally important due to its wide distribution in flowering plants [53]. In *Arabidopsis*, mutations in domain II leaded to enhanced auxin responses by blocking the degradation of IAA proteins [54]. However, in MtIAA protein family, some MtIAAs did not contain domain II. It suggested that the degradation of these MtIAA proteins were independent on domain II- mediate auxin responses. Most of the MtIAA proteins contain domain III–IV, which are the important domains for binding to ARFs. Specially, MtIAA11 does not contain domain II and MtIAA14 only contains a partial domain IV.

### Auxin regulated gene expression

In the promoters of most auxin-responsive genes, auxin-responsive (AuxRE) *cis*-elements are characteristic structures [8]. Promoter analysis illustrated that most promoters of *MtIAA* genes contain AuxRE *cis*-elements or their variants (Figure 6) [55]. Our analysis showed that the motifs for AuxREs, ZREs and MREs related elements were significantly enriched in the promoters of *MtIAA* genes. All the promoters of *MtIAA* genes contained AuxRE *cis*-elements or their variants, expect for the promoter of *MtIAA13*, which did not contain any AuxRE *cis*-elements. Furthermore, take the classical AuxRE *cis*-elements AUX1 and AUX2 as examples. Ten of the seventeen *MtIAA* gene promoters contained more than one AUX1 or AUX2 within 1000 bp before ATG. It was indicating the expressions of *MtIAA* family genes could be regulated by auxin signaling.

In *M. truncatula*, six MtIAA genes were down-regulated by IAA treatment in shoot and 7 MtIAA genes were reduced by IAA treatment in root. The *MtIAA* family genes showed different expression patterns to response to exogenous auxin stimulation. Interestingly, the changes of expression levels of 5 *MtIAA* genes in shoot and 6 *MtIAA* genes in root were maintaining stability over time while the rest of *MtIAA* genes continuously increased or decreased expressions during the time course. The dynamic expressions of *MtIAA* family genes under IAA treatment indicated that different *MtIAA* genes are involved in the variability of auxin regulation.

To gain insight into the spatial pattern of the expressions of *MtIAA* genes, their transcript accumulations were analyzed in different plant tissues and organs. The clustering revealed five main clades: A, B, C, D and E. Most of the *MtIAA* family genes displayed the highest expression in roots. Interestingly, only *MtIAA9* showed higher expression level in flower. No correlation was found between the clustering based on phylogenetic analysis and gene expression patterns. Some *MtIAA* genes displayed obvious preferential expression in a specific tissue suggesting the regulation of these genes might function essentially at the post-translational level [34]. Overall, the root-preferential expression of *MtIAA* family genes is indicative of their involvement in root developmental processes and responses to environmental stimulus.

### MtIAAs were involved in nodule formation during the early phase of *Sinorhizobium meliloti* infection

Nitrogen-fixing nodule is the essential organ for symbiotic interactions between legumes and rhizobia. Formation of nodule helps soil rhizobia to convert atmospheric N_2_ into ammonia for *M. truncatula* absorption [56]. Rhizobial infection, which leads to signal exchanges between the hosts and the bacteria, is the first step for nodule organogenesis and many transcriptome and proteome changes occur in both the shoots and roots during the early stage of rhizobial infection [46], [57]. Auxin plays an important role in the initiation and development of nodules of different legumes, including white clover, *Lotus japonicas* and *M. truncatula* [57], [58]. A *Lotus japonicus* mutant *rel3* exhibited insensitivity to auxin and produced fewer nitrogen-fixing nodules [56]. Here, we used *M. truncatula* as a model indeterminate legume to study how Aux/IAA-mediated auxin signaling is involved in nodule formation during the early phase of *S. meliloti* infection.

Auxin regulates down-stream genes transcription by promoting the degradation rate of the Aux/IAA family proteins, which function as transcriptional repressors [59]. The rhizobial-regulation expressions of *MtIAA* family genes trigger physiological responses in a spatio-temporal specific manner during the formation of nodules. Our qRT-PCR data showed that most *MtIAA* genes were involved in the inoculation of roots with the nodulating symbiont (*S. meliloti*). The expression profiles of *MtIAA* genes changed significantly during *S. meliloti* infection and MtIAA-mediated auxin signaling may activate or suppress the functions of many down-stream genes involved in the formation of nodules. Inoculation of *M. truncatula* roots reduces endogenous auxin loading from shoots to roots [60]. Most of *MtIAA* family genes were down-regulated in roots by *S. meliloti* infection and up-regulated in shoots. *S. meliloti* infection may suppress the MtIAA-mediated auxin signaling in roots by controlling auxin relocation between shoots and root system. Our results revealed that the changes of expression levels of *MtIAA* family genes occurred at early time points after *S. meliloti* infection (Figure 7). The expression of most *MtIAA* genes was found to be involved in the inoculation of roots with *S. meliloti*, which suggests a putative role in the formation of nitrogen-fixing nodules. In comparison to mock inoculation, MtIAA-mediated auxin signaling may activate or suppress the down-stream genes involved in the formation of nodules under *S. meliloti* infection.

Long distance of auxin polar transport between shoots and roots is the essential process for formation of nodules in *M. truncatula* [60]. Deviant nodules that lack vascular strands or with proliferating vascular tissue could be induced by auxin transport inhibitors like naphthylphthalamic acid (NPA) [33], [61]. It suggested that polar auxin transport plays a central role in vascular bundle formation in nodules. In this work, an auxin influx inhibitor 1-NOA [62], was used to interfere with MtIAA-mediated auxin signaling during the early phase of *S. meliloti* infection. To test how MtIAA-mediated auxin signaling plays a role in nodule formation during the early phase of *S. meliloti* infection, we analyze the qRT-PCR data of *MtIAA* genes expression levels under –*Sin*/−NOA, +*Sin*/−NOA, −*Sin*/+NOA and +*Sin*/+NOA four treatments respectively. The expression profiling of *MtIAA* family genes did not change too much in both shoots and roots under 1-NOA treatment only (−*Sin*/+NOA). 1-NOA treatment (+*Sin*/+NOA treatment) reduced the differences in *MtIAA* gene expression between shoots and roots compared to +*Sin*/−NOA inoculated plants. *S. meliloti* infection has a significant effect on the MtIAA-mediated auxin signaling pathway. However, inhibition of auxin transport relieved the differences in expressions of *MtIAA* genes between roots and shoots during the early phase of *S. meliloti* infection. *S. meliloti* infection first triggered the MtIAA-medicated auxin signaling to help nodule initiation and differential MtIAA-mediated auxin signaling between shoots and roots may be an essential process for expression regulation of nodule-related downstream genes.

## Conclusion

*M. truncatula*, a close relative of alfalfa, is a model legume widely used in nitrogen fixation, symbiosis and legume genomics studies [63]. In summary, the detailed analysis of *MtIAA* family genes provides new insights into the structure and expression of this gene family that plays an important role in auxin signaling and gene expression regulation of *M. truncatula* under different conditions. The involvement of *MtIAA* genes during the early phase of *S. meliloti* infection helps us to understand the role of auxin signaling in the regulation of nodule formation.

## Supporting Information

Table S1 **The qRT-PCR primers for** ***MtIAA*** **family genes.**
(XLS)

Table S2 **The location information of AuxRE** ***cis*** **-elements in** ***MtIAA*** **family gene promoters.**
(XLS)

Table S3 **The information of** ***AtIAA*** **gene family.**
(XLSX)
